# The Need of More Facilities for Post-Graduate Study

**Published:** 1915-09-18

**Authors:** 


### POST-GRADUATE EDUCATION AND THE WAR.

### The Need of More Facilities for Post-Graduate Study.

It is convenient to regard post-graduate education as falling under three headings. The first refers to the education and training which a young qualified man receives during his tenure of hospital appointments, whether as clinical assistant in the out-patient department or as house physician or house surgeon; the second relates to studies the object of which is the passing of examinations for the higher diplomas, such as F.R.C.S. or M.R.C.P.; while the third heading includes organised instruction in the scientific side of medicine and surgery after the stage of examinations and hospital appointments is past. Whereas the two former headings refer to post-graduate education in well-established forms, the last is somewhat in the nature of an unrealised ideal. As regards the first heading, to the newly qualified doctor the house appointments and the out-patient posts at a large general hospital are of the greatest possible educational value, since they offer opportunities for examining and treating a great variety of cases under the supervision of an experienced physician or surgeon. In normal times these appointments are much sought after, so that generally there are numerous applicants for each post, and only the more able candidates stand a chance of success. It is indeed unfortunate that, of all the men who become qualified doctors, it falls to the lot of only a few to obtain the unique experience and training which these posts afford.

### The Value of Hospital Experience.

The war has made havoc of this scheme of hospital appointments. Far from there being a choice of selection, hospital authorities cannot get a sufficient number of qualified men of any type to fill the posts. The reason for this, of course, lies in the fact that nowadays the chief aim of the medical student is to get qualified as soon as possible, and to join one of the Services. In the first excitement of the war this rush to join the Navy or Army placed the hospitals in serious difficulties. Now there is evidence of a more sober judgment. The newly qualified man realises that it is beneficial not only to himself, but also to the hospital and to the Service which he proposes to join, to gain even three months' practical experience in a hospital appointment. In many instances hospitals have reduced the tenure of office from six months to three months, and the War Office has recently given facilities for young doctors to receive commissions, while remaining at work at a hospital until required.

### Higher-grade Examinations.

The second category of post-graduate education, which concerns the passing of some higher-grade examination, is also much affected by the war. The number of candidates presenting themselves for these more advanced degrees has greatly diminished. At one large hospital, classes for the Surgical Fellowship examination, which have been held regularly for years past, have been abandoned owing to the lack of candidates. So far reference has been made only to the more ordinary forms of post-graduate education. But the term "post-graduate education" has in recent years acquired somewhat of a special significance. It does not mean simply education in general subsequent to graduation, but it conveys the idea of a continuance of the organised and systematised study of medicine and surgery without any ulterior motive of examination.

### Facilities for Post-Graduate Study Needed.

The training of the English medical student is largely clinical, and it is admitted that the English general practitioners are of a higher standard than the practitioners of any other country. Nevertheless opportunities after qualification for the specialised study of the various departments of medicine would undoubtedly be beneficial. Various attempts have been made to facilitate this study, but, so far, with only a limited degree of success; and the war has added to the hindrances. There are many reasons for the failure, chief of which are the inherent traits of the English race, and the sharp line of demarcation which tradition has drawn between the general practitioner and the consultant. Again, the student, after spending perhaps six years in becoming qualified and two years in hospital appointments, considers that he has spent enough time and money on education. He has satisfied his conscience that he can go forth into the world and profess to treat the sick, without the sick running any unfair risks. Why should he pursue further his scientific studies? He cannot hope to advance science, and when he feels out of his depth he can always call in a consultant.

### The Effect of the War.

For such reasons as these post-graduate educa tion has never really flourished in this country, though earnest efforts have made the beginning of it a matter of hope. There can be no denying the fact that post-graduate education in all its aspects has been sadly wrecked by the war. The clearing of the site may in the end prove beneficial, and new efforts may start with the advantage of experiences gained from the past. It would, for instance, be an advantage if the experience which hospital appointments afford could be extended to greater numbers and not restricted to the more competent few. Moreover any system for the furtherance of the scientific study of medicine, and for the bridging over of the great gulf fixed between the practitioner and the consultant, could not but be beneficial in its results.

